# Werbung oder Information in der Ophthalmologie? Wissenschaftliche Bewertung einer YouTube-Stichprobe

**DOI:** 10.1007/s00347-020-01105-6

**Published:** 2020-04-30

**Authors:** Kilian Schrenk, Ramin Khoramnia, Nicolas Feltgen, Werner Bachmann, Focke Ziemssen, Jens Martin Rohrbach, Spyridon Dimopoulos

**Affiliations:** 1 Department für Augenheilkunde, Eberhard-Karl Universität Tübingen, Elfriede-Aulhorn-Str. 7, 72076 Tübingen, Deutschland; 2 Universitätsaugenklinik Heidelberg, 69120 Heidelberg, Deutschland; 3 Augenklinik der Universitätsmedizin Göttingen, Göttingen, Deutschland; 4 ReVis Augenklinik Aschaffenburg, Aschaffenburg, Deutschland

**Keywords:** Werbung, Marketing, Leistungsspektrum, Gesundheitskompetenz, Information, Berufsrecht, Heilversprechen, Komplikationen, Information, Health literacy, Advertisement, Marketing, Performance spectrum, Professional law, Treatment promise of cure, Complications

## Abstract

**Hintergrund:**

Trotz der Liberalisierung des Heilmittelwerbegesetzes ist anpreisende, irreführende oder vergleichende Werbung nach wie vor berufswidrig. Angesichts des zunehmenden Engagements von Finanzinvestoren und der Ökonomisierung in der Augenheilkunde sollte diese Arbeit prüfen, welche Aussagen in offen zugänglichen Videos der YouTube-Plattform getroffen werden.

**Methode:**

Mit den Suchbegriffen „Augenarzt“, „Augenärztin“ und „Augenzentrum“ sowie vordefinierten Kriterien (deutsche Herkunft, Audiospur mit Text, Dauer >1 min) wurden Videos identifiziert und in eine anonymisierte Textform transkribiert. Mittels eines eigens entwickelten Fragebogens wurden die Einzelaussagen daraufhin einer kritischen Prüfung durch jeweils 3 Experten unterzogen sowie Klarheit, Relevanz und Vollständigkeit bewertet. Parallel wurden Laien abgefragt, wie überzeugend, verständlich und vollständig sie die Informationen bewerten und ob sie sich von den Ärzten behandeln lassen würden.

**Ergebnisse:**

Von 68 Videos erfüllten 30 die definierten Einschlusskriterien; 46 % der Videos thematisierten Verfahren der refraktiven Chirurgie. Aus Sicht der Experten waren mindestens 11,8 % der Einzelaussagen vollständig falsch oder wenig korrekt. Über 80 % der Filme stellten Informationen unvollständig dar. So wurden z. B. peri- und postoperative Komplikationen nur von 3 Filmen angesprochen. Laien bewerteten die Texte recht uneinheitlich und konnten nicht die Videos identifizieren, die aus Sicht der ophthalmologischen Fachärzte problematische Aussagen enthielten. Es wurden Konflikte mit den rechtlichen Anforderungen an Werbung z. B. in der Verwendung entsprechender Superlative festgestellt. Eine ausgewogene Darstellung wie Alternativen zu den Behandlungsverfahren war kaum enthalten, die Verständlichkeit für Laien war verbesserungswürdig.

**Schlussfolgerungen:**

Nur eine geringe Anzahl frei zugänglicher Videos bot aktuelle, wissenschaftlich fundierte und korrekte Informationen. Bisher berücksichtigen nur wenige Augenärzte die juristischen und moralischen Anforderungen an werbende Aussagen. Daher ergeben sich möglicherweise negative Auswirkungen auf das Berufsbild in der Öffentlichkeit, und Chancen zur Gesundheitsförderung bleiben ungenutzt.

## Abstract (en)

**Background:**

Despite the liberalization of the Therapeutic Products Advertising Act, advertising that praises, misleads or compares is still contrary to the German professional conduct. In view of the increasing commitment of financial investors and the economization in ophthalmology, this study was initiated to examine and check which (advertising) statements are affected in openly accessible videos on the YouTube platform.

**Methods:**

Using the search terms “ophthalmologist” and “eye center” as well as predefined criteria (German origin, audio track with spoken text, duration >1 min), videos were identified and transcribed into an anonymized text version. Using a self-developed predefined questionnaire, the individual statements were then subjected to a critical review by three experts and clarity, relevance and completeness of the information were also assessed. At the same time, laypersons were asked how convincingly, comprehensibly and completely they assessed the information and whether they would want to be treated by the physicians.

**Results:**

Out of 68 videos 30 met the defined inclusion criteria. Of the videos 46% focused on procedures for refractive surgery. From the experts’ point of view, at least 11.8% of the individual statements were completely wrong or only slightly correct. Over 80% of the videos presented information incompletely. Perioperative and postoperative complications were only mentioned by three films. Laypersons evaluated the texts very inconsistently and could not identify the videos that contained problematic statements as assessed by the ophthalmologists. Conflicts with the legal requirements for advertising, e.g. the use of lurid superlatives were found. A balanced presentation, such as alternatives to treatment procedures was rare. There was a great need to improve the comprehensibility for laymen.

**Conclusion:**

Only a very small number of freely accessible videos provide scientifically founded and correct information. Until now, many ophthalmologists do not sufficiently consider the legal and moral requirements for advertising statements. Therefore, there may be negative effects on the professional image in the public eye and opportunities for health promotion remain unused.

Verbote des Heilmittelwerbegesetzes sind in den vergangenen Jahren immer weiter liberalisiert worden [1]. Inzwischen lassen die gesetzlichen Rahmenbedingungen deutlich mehr Spielräume (Abb. 1): Während im Rahmen der Arzneimittelwerbung Angst- und Bangemachen verboten blieb, wurde für Heilberufe das generelle Verbot aufgehoben, mit Angstgefühlen zu werben. Genauso sind mittlerweile Anleitungen zulässig, mit denen Betroffene bestimmte Krankheiten, Leiden, Körperschäden oder krankhafte Beschwerden an sich selbst erkennen können.

**Figure 1:**
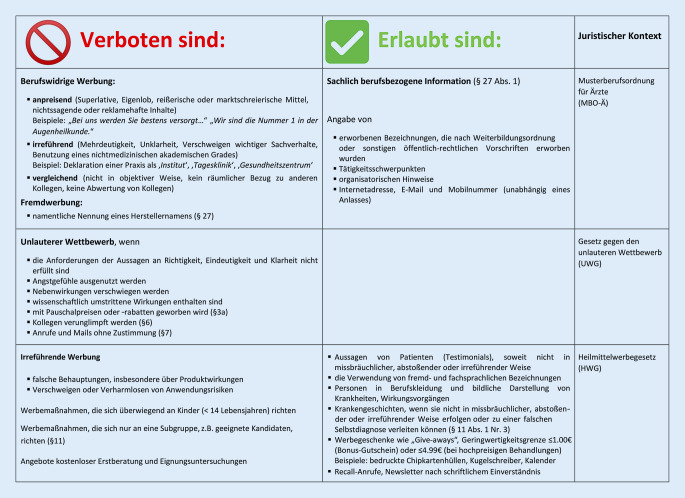

Während die sachliche Information im Hinblick auf das Patientenwohl und die Patientenautonomie hier durchaus erwünscht sein kann, ist anpreisende, irreführende oder vergleichende Werbung aber nach wie vor berufswidrig. Eine Angabe ist irreführend, wenn sie dazu geeignet ist, den angesprochenen Adressaten einen unrichtigen Eindruck zu vermitteln. Dabei spielt es keine Rolle, ob es tatsächlich zu einer Irreführung kommt. Die Ärztekammer hat in ihrer Bekanntmachung 2017 darauf hingewiesen, dass eine dem Selbstverständnis der Ärzteschaft zuwiderlaufende Kommerzialisierung des Arztberufes durch Werbung vermieden werden soll [1]. Zuständige Landesärztekammern erlangen aber nicht zwingend Kenntnis von Verletzungen der Berufspflichten, zumal es kaum aktive Ermittlungen oder Aktivitäten der Strafverfolgung gibt. Daher stellt sich die Frage, wie ausgerechnet die adressierten Laien differenzieren wollen, welche Behauptungen inhaltlich korrekt sind und wo die Grenzen der sachlichen berufsbezogenen Informationen verlassen werden.

Unzulässig sind unklare Bezeichnungen, die z. B. mit schwieriger zu erwerbenden Fähigkeiten verwechselt werden könnten, wie auch Vergleiche mit der Konkurrenz. Mehrdeutigkeit, Unklarheit, Verschweigen wichtiger Sachverhalte können genauso irreführen wie die Benutzung eines nichtmedizinischen akademischen Grades oder die Deklarierung als „Institut“ oder „Zentrum“, wenn die Voraussetzungen nicht vorliegen (BVerfG, 07.03.2012, Az.: 1 BvR 1209/11). In der Augenheilkunde sind inzwischen zahlreiche Finanzinvestoren engagiert, die nicht selten regionale Satellitenstrukturen bilden [4]. Werbende Aussagen sind v. a. im Bereich der Selbstzahlerleistungen verbreitet, die letztlich eine Kundengewinnung und Patientenselektion zum Ziel haben: Während um lukrativere Behandlungsfälle wie refraktive Operationen, Kataraktchirurgie und intravitreale Injektionen geworben wird, wurden Unterversorgung und Zugangshürden für Patienten mit Glaukom, Kinder oder Pflegegrad berichtet [5], für die es somit schwieriger werden kann, eine entsprechende Betreuung zu erfahren oder Termine zu vereinbaren.

Allein in den USA sind die Ausgaben für das Marketing von Medikamenten, Aufklärungskampagnen und Gesundheitsdiensten bis 2016 auf 30 Mrd. Dollar angestiegen [6]. Seit 1997 ist die direkt an den Endverbraucher gerichtete Werbung (DTC) am schnellsten gewachsen. Die American Academy of Ophthalmology (AAO) hat allerdings klare Vorgaben zu Äußerungen in der Öffentlichkeit in einem *Code of Ethics* explizit geregelt [7]: Mitglieder dürfen keine „*false, untrue, deceptive, or misleading information through statements, testimonials, photographs, graphics or other means*“ abgeben. Abgesehen davon, dass es in Einzelfällen schwierig sein kann, einen einheitlichen Konsens über die Grenzen herzustellen, wann es sich um eine Fehlinformation handelt, haben die augenärztlichen Organisationen in Deutschland bisher keinen vergleichbaren Codex formuliert, der eine standardisierte Herangehensweise mit konkreten Konsequenzen und Sanktionen formaljuristisch regeln und somit erst ermöglichen würde. Aus moralischer Sicht dürften Informationen im Internet – auch angesichts der zeitlich und räumlich uneingeschränkten Sichtbarkeit – nicht trügerisch sein, dürften keine Ängste oder Schwachstellen Betroffener adressieren oder ungerechtfertigte Erwartungen schüren, sondern müssten eine realistische Bewertung von Risiko, Netto-Nutzen und Alternativen ermöglichen [8]. Dabei sollten die Informationen, soweit sie wahr und sachgerecht sind, verständlich für den Patienten dargebracht werden [1].

In anderen Fachrichtungen haben Beratungsfirmen aktiv für ein stärkeres Online-Marketing geworben; Juristen haben auf die verschobenen Grenzen der Anforderungen aufmerksam gemacht [9–11]. Die Regeln unterscheiden sich zudem erheblich zwischen einzelnen Ländern [12]. Obwohl für einige Disziplinen Stichproben schon einmal Werbung und Online-Inhalte analysiert haben [13–15], war nicht bekannt, wie deutsche Augenärzte Videos als Informationsmedium nutzen. Im Rahmen dieser wissenschaftlichen Arbeit sollten daher Filme aus der Augenheilkunde identifiziert und in anonymisierter Weise analysiert und bewertet werden. Bewegte Bilder sind nicht zuletzt mit dem Aufstieg der sozialen Medien ein immer beliebterer Weg, Informationen zu transportieren, medizinische Sachverhalte zu erklären oder das eigene Leistungsangebot zu präsentieren.

## Material und Methode

Die Suche auf dem YouTube-Portal (https://www.youtube.com/) erfolgte nach einem vordefinierten Protokoll. Einschlusskriterien waren die Verfügbarkeit am 14.08.2019 unter den Begriffen „Augenärztin“ (*n* = 1), „Augenarzt“ (*n* = 26) und „Augenzentrum“ (*n* = 31). Ausgeschlossen wurden alle Videos mit einer Dauer unter 1 min oder über 4 min Dauer sowie eine Audiospur ohne Text. Die Beschränkung auf die Zeit erfolgte, weil es gesicherte Erkenntnisse über das Konsumverhalten gibt: Für die gängigen Internetplattformen wurde ein unmittelbarer Zusammenhang zwischen der Länge eines eingestellten Videos und der relativen und absoluten Betrachtungsdauer durch die Konsumenten gefunden [16]. Außerdem wurden keine Videos berücksichtigt, die außerhalb von Deutschland eingestellt wurden (*n* = 10) oder deren Urheber ein Unternehmen wie eine Medienanstalt oder nichtärztlicher Autor, z. B. Privatpersonen, war.

Für die verbliebenen 30 Videos wurden die inhaltlichen Kategorien (Privatpraxis, Sonderleistungen) evaluiert. Unabhängig von den verwendeten Bildformaten, die von einer klassischen Interviewsituation über die Beschreibung eines Behandlungsverlaufs bis zu grafischen Animationen reichten, wurden die gesprochenen Texte der Audiospur in eine verschriftlichte Form überführt. Die Inhalte wurden durch das Entfernen von Personen- und Ortsnamen anonymisiert. Nach der Zuordnung (allgemeine Augenheilkunde, refraktive Chirurgie, vitreoretinale Chirurgie und Retinologie) erfolgte eine verblindete Bewertung der Gesamttexte und definierter Einzelaussagen mittels eines Fragebogens, der eine Auswahl nach Likert-artigen Kategorien vorsah. Es handelt sich hierbei um Aussagen, denen die Befragten auf einer vorgegebenen mehrstufigen Antwortskala zustimmen. In der Pilotierung der formal ordinal- bzw. rangskalierten Items wurde durch Symmetrie und grafische Darstellung das Ziel einer äquidistanten Abstufung unterstützt. Aus dem Pool der Experten erfolgte aber auch eine freie unvoreingenommene Kommentierung. Neben der Plausibilität wurden die Klarheit des Textes, die Relevanz der Informationen und die Vollständigkeit bewertet. Für den Gesamttext wurde außerdem abgefragt, ob Komplikationen oder Nebenwirkungen der genannten Behandlungsverfahren oder Medizinprodukte angesprochen oder genannt wurden. Die sachliche Richtigkeit der Einzelaussagen konnte auf einer 5‑stufigen Skala („vollständig falsch“, „wenig korrekt“, „teilweise korrekt“, „weitestgehend korrekt“, „korrekt“) bewertet werden, anpreisende, irreführende, mehrdeutige oder vergleichende Aussagen konnten markiert werden.

Ähnlich wie die Bewertung durch Fachärzte erfolgte die Evaluation durch medizinische Laien in randomisierter Reihenfolge der Texte. Um eine möglichst unabhängige und homogene Bewertung durch Laien ohne medizinischen Hintergrund zu erreichen, wurden Studenten ohne medizinischen Hintergrund (Familie, Studium) angesprochen. Die Laien wurden nach ihrer Einschätzung gefragt, ob Ärztin/Arzt/Arztpraxis überzeugend wirke, die Informationen verständlich und vollständig seien. Außerdem wurde erfragt, ob sie sich selbst dort behandeln lassen würden. Außerdem wurden die freien Kommentare ausgewertet und offene Fragen erfasst. Um eine möglichst vollständige Maskierung zu erreichen, wurde sowohl bei Experten als auch Laien darauf geachtet und dazu aufgefordert, wegen der beabsichtigten Verblindung keine eigenen Recherchen im Internet zu betreiben.

Für die formalen Parameter wurden die Texte nach Umfang, Zugriffsraten im Netz, Anzahl der Wörter und Sätze sowie der Verwendung von Superlativen und Herstellernamen analysiert. Die deskriptive Statistik erfolgte mit JMP 14.2.0 (SAS Institute). Neben einfachen Korrelationsanalysen wurde Multi-Rater Kappa nach Fleiss als Maß der Übereinstimmung der abgegebenen Bewertungen verwendet [17].

## Ergebnisse

Von den identifizierten 30 Videos behandelte die Mehrheit Inhalte der refraktiven Chirurgie (*n* = 13, LASIK, Presbyopie, Sonderlinsen) oder allgemeine Inhalte (*n* = 13, trockenes Auge, Glaukom, Kontaktlinsen, Diagnostik, Orthoptik). Nur ein kleiner Anteil (*n* = 4, Glaskörpertrübungen, Netzhautchirurgie, Netzhautlaser) wurde dem Bereich der vitreoretinalen Chirurgie zugeordnet. Die Urheberschaft lag bei insgesamt 20 verschiedenen Arztpraxen, von denen 4 rein privatärztlich tätig waren. Die große Mehrheit der Stichprobe verantwortete nur ein einzelnes Video, nur 3 Ärzte hatten offensichtlich mehrere Videos unterschiedlicher Inhalte (*n* = 2, 4 oder 7) eingestellt.

Die Filme hatten im Median eine Dauer von 130 s (Spanne: 67–233 s). Die Videos waren im Median seit 30 Monaten im Netz (Spanne: 6 bis 99 Monate, 95 %-KI: 25 bis 46 Monate). Die zugehörigen Arztpraxen waren in der Mehrheit in Kernstädten (*n* = 16) oder im verdichteten Umland (*n* = 12) angesiedelt, in denen die Arztdichte über der durchschnittlichen Anzahl an Augenärzten im Bundesvergleich lag. Eine Minderheit der Ärzte (*n* = 12) arbeitete an nur einem Standort, 3 hatten sogar mehr als 10 Standorte. Tätig waren im Median 5 Ärzte (Spanne: 1 bis 40). In Bezug auf die Aufrufe (Median: 1027, Spanne: 21 bis 9527) gab es in der Stichprobe keinen offensichtlichen Zusammenhang zwischen Umfang und Zugriff. Lediglich die absolute Zahl der Aufrufe zeigte Assoziation mit der zeitlichen Verfügbarkeit im Netz (Pearson-Koeffizient: 0,45, *p* = 0,013). Der relative Zugriff, d. h. Aufrufe pro Monat, zeigte nur einen Zusammenhang mit dem Ort der Praxis: Die Videos von Ärzten im ländlichen Raum wurden im Mittel mit 18,9-mal pro Monat deutlich seltener aufgerufen als die der Siedlungstypen mit höherer Bevölkerungsdichte (Umland: 68,0 bzw. Kernstädte: 71,2). Im Median wurden die Videos jeweils 49-mal pro Monat aufgerufen.

### Bewertung durch Experten

**Figure 2:**
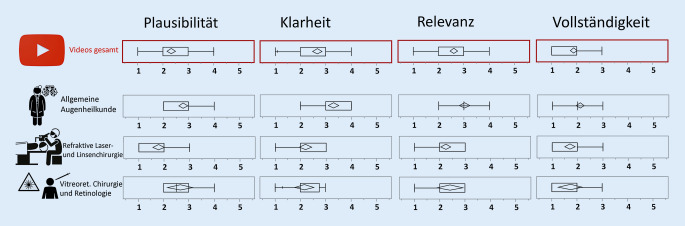

In Bezug auf die Plausibilität sahen die Experten bei mehr als einem Drittel der Videos viele oder sehr viele widersprüchliche Aussagen oder Denkfehler (Abb. 2). Keines der Videos wurde von mehr als 2 Gutachtern als völlig frei von Widersprüchen oder Fehlern gesehen. Die Interrater-Übereinstimmung war hier moderat (κ 0,423 nach Fleiss).

Zwischen 50 und 70 % der Videos wurden in Bezug auf die Klarheit der Inhalte als für Laien zumindest teilweise verständlich bewertet (κ 0,533 nach Fleiss). Die Gutachter hielten zwischen 6,7 und 13,3 % der Texte für vollständig unklar. Dabei wurden die angesprochenen Inhalte mehrheitlich für relevant gehalten, obwohl es hier eine gewisse Heterogenität in Bezug auf einzelne Themen gab (κ 0,458 nach Fleiss); 6,7–10 % der angesprochenen Themen wurden für völlig irrelevant gehalten. Die größte Übereinstimmung in der Bewertung (κ 0,599) gab es aber in der Einschätzung der Vollständigkeit der Informationen. Zwischen 83,3 und 86,6 % der Videos wurden in der Beurteilung des Informationsgehalts für unvollständig gehalten. Die Gutachter identifizierten nur 3 Videos, in denen vereinzelt eine Art von Nebenwirkung oder mögliche Komplikation der diskutierten Behandlungsverfahren angesprochen wurde, das Thema evtl. Nachteile blieb in allen Videos ausgespart.

### Sachliche in/korrekte Aussagen

Aufgrund unterschiedlich langer Texte bzw. des Umfangs und der Länge der Sätze wurde eine unterschiedliche Anzahl an Aussagen pro einzelnem Video beurteilt (Mittelwert: 9, Spanne: 3 bis 21 Aussagen). Allerdings sahen sich die Experten für einen Teil der insgesamt 278 Aussagen nicht in der Lage, den Wahrheitsgehalt zu beurteilen, weil Themen wie Berufserfahrung, berichtete Operationszahlen, angegebene Ausstattung oder das Angehören von Fachkommissionen ohne Kenntnis der Namen und örtlichen Gegebenheiten nicht bewertet werden konnten. Daher konnten 213 (25,6 %) der theoretisch möglichen 834 Bewertungen von Aussagen nicht erfolgen.

**Figure 3:**
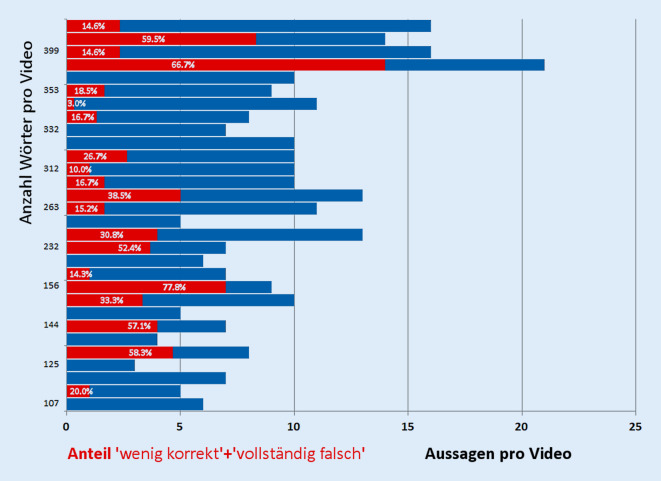

Es gab einen gewissen Anteil an Aussagen, die in der Begutachtung einheitlich als „vollständig falsch“ oder „wenig korrekt“ bewertet wurden (Abb. 3); 33 (11,8 %) der gesamten Aussagen wurden in Übereinstimmung dieser Kategorie zugewiesen. Hierbei muss aber berücksichtigt werden, dass die Experten in der unabhängigen Einordnung auf der 5‑stufigen Likert-Skala insgesamt nur leidlich (κ 0,285 nach Fleiss) in der genauen Gewichtung des Wahrheitsgehalts einzelner Aussagen übereinstimmten. Jeder einzelne Gutachter bewertete einen Anteil zwischen 24,1 und 28,1 % der Aussagen als „falsch“ oder „wenig korrekt“. Obwohl jedes bewertbare Video mindestens 1 Aussage enthielt, die als „teilweise“ oder „weitestgehend korrekt“ angesehen wurde, gab es nur 4 Videos, 3 davon aus dem Bereich der allgemeinen Augenheilkunde, deren Aussagen ausschließlich als mindestens „teilweise korrekt“ bewertet wurden. Mindestens 2 der Urheber mehrerer Videos mit Auffälligkeiten des Wahrheitsgehalts gehörten der Gruppe mit vielen Standorten und mehreren Videos an, für die eine eigentümergeführte Organisationsstruktur nicht unbedingt vorausgesetzt werden darf.

Zwischen 34,9 und 56,8 % der Aussagen wurden als „anpreisend“ eingeordnet. „Irreführend“ bewertete jeder einzelne Gutachter zwischen 25,2 und 27,1 % der Feststellungen. „Mehrdeutig“ waren immerhin noch 13,3–19,4 % der Textpassagen, während „vergleichende“ Aussagen selten identifiziert wurden (8,3–15,5 %). Allerdings hielten die Experten auch nur 9,7–19,1 % der Feststellungen für nachvollziehbar.

Die Tab. 1 enthält einzelne Beispiele, die angesichts der Bewertungen für problematisch gehalten werden. Irreführend im Sinne nach § 3 Nr. 1 HWG erachteten die Experten z. B. Aussagen zu einer Lasertherapie, der bestimmte, noch nicht gesicherte Wirkungen (Verhinderung einer neovaskulären Makuladegeneration) oder eine allgemeine therapeutische Wirksamkeit zugesprochen wurden, die sie tatsächlich nicht haben oder die nicht erwiesen sind.

**Table 1:** 

*Aussagen *(≥2 Bewertungen „wenig korrekt“ oder „vollständig falsch“)
„*Der 2RT-Laser ist ein neuer revolutionärer Ansatz zur Behandlung der altersabhängigen Makuladegeneration, der AMD.*“
„*Mit diesem Laser können wir nun zum ersten Mal mit sanften Nanolaser-Impulsen das retinale Pigmentepithel so stimulieren, dass über eine Immunantwort des Körpers Ablagerungen, die aufgrund von Durchblutungsstörungen entstanden sind, abgebaut werden können*.“
„*Damit können wir den Übergang in eine feuchte und damit die finale Form der AMD verhindern*.“
„Neuerdings gibt es einen Laser, einen sog. Floater-Laser, – Floater ist nämlich auch ein Name dafür – der diese Teilchen, diese Trübungen berührungslos auflösen kann.“
„*Die Glaskörpertrübungen sind danach komplett verschwunden*.“
„*Die Premiumlinse hat einen verbesserten UV-Filter, sodass Ihr Sehzentrum geschützt wird*.“
„*Die meisten Patienten entscheiden sich derzeit für eine Premiumlinse*.“
„*Beim Laser können fast alle Schritte, die vorher der Operateur manuell durchführen musste, nun mit dem Laser besonders sicher und extrem präzise durchgeführt werden*.“
„*Ich denke, der sollte mindestens 1000 Vitrektomien dann durchgeführt haben und auch über 800 Vitrektomien im Jahr machen, dass er einfach diese Routine hat, dass er die Erkrankung, die Sie haben, dann am besten dann auch therapieren kann*.“
„*Ab 18 ist das ideale Alter. Hier ist es sogar gut, sich früh behandeln zu lassen, weil die Behandlung der Kurzsichtigkeit das Fortschreiten der Kurzsichtigkeit stoppen kann*.“
„*Wenn nun die trübe Linse durch eine künstliche Linse ersetzt wird, dürfen Sie dabei zwei Entscheidungen treffen*:“
„*Sie wird vor allem so früh wie möglich durchgeführt, d. h. nach der ersten Diagnose einer Linsentrübung*.“
„*Der Laser kann zudem die Linse so zentral auf den Mikrometer genau platzieren, dass für Sie die Sehschärfe hinterher nochmals deutlich besser wird*.“
„*Das Verfahren ist sehr, sehr schnell. Wir brauchen ungefähr sieben Minuten. In dieser Zeit sind beide Augen behandelt*.“
„*Die ICL bringt auch keine neuen Fehler ins Auge, was z. B. bei einer Korrektur mittels Laser schon der Fall sein kann, dass durch den Abtrag sich die Hornhaut verändert oder über die Jahre verzieht und dadurch mehr Fehler entstehen*.“
„*Damit sind wir 25 × genauer als wir das mit Kontaktlinsen und Brillen sein können*.“
„*Wir verbessern damit das Kontrastsehen*.“
„*Diese Linse wird individuell hergestellt und ist dementsprechend auch austauschbar und deshalb ist da auch ein höherer Preis veranschlagt*.“
„*Die moderne FEMTO-LASIK kann mehr als nur die Brillenkorrektur. Sie kann auch die Qualität des Sehens verbessern, beim Kontrastsehen, beim Nachtsehen, beim 3‑D-Sehen*.“
„*Ich bin selbst gelasert und was uns besonders freut, dass immer mehr Ärzte und besonders Augenärzte dieser Technik vertrauen*.“
„*Statistisch kommt ein grauer Star ab einem bestimmten Alter folglich bei fast allen Menschen der Welt vor*.“

### Bewertung durch Laien

Jeder Text eines Videos wurde auch von jeweils 3 Laien bewertet. Insgesamt wiesen die Bewertungen eine große Heterogenität auf: Die Kategorien wiesen daher allenfalls eine schwache Übereinstimmung nach Landis und Koch auf [18]. In dem Gesichtspunkt, wie überzeugend sie den Text bewerteten, stimmten die Laien-Beobachter kaum überein (Fleiss’ κ 0,005). Die Items „Verständlichkeit“ (Fleiss’ κ 0,142) und „Vollständigkeit“ (Fleiss’ κ 0,103) wiesen eine minimal stärkere Übereinstimmung auf. Die Frage, ob sich die Personen von den Ärzten bzw. der Arztpraxis beraten und behandeln lassen würden, wurde von den Laien für das identische Video wiederum recht unterschiedlich beantwortet (Fleiss’ κ 0,050), allerdings zeigte sich jeweils ein starker Zusammenhang zwischen der empfundenen Überzeugungskraft eines Filmtexts und dem geäußerten Vertrauen gegenüber dem Urheber (Koeffizient 0,84, *p* < 0,001).

**Figure 4:**
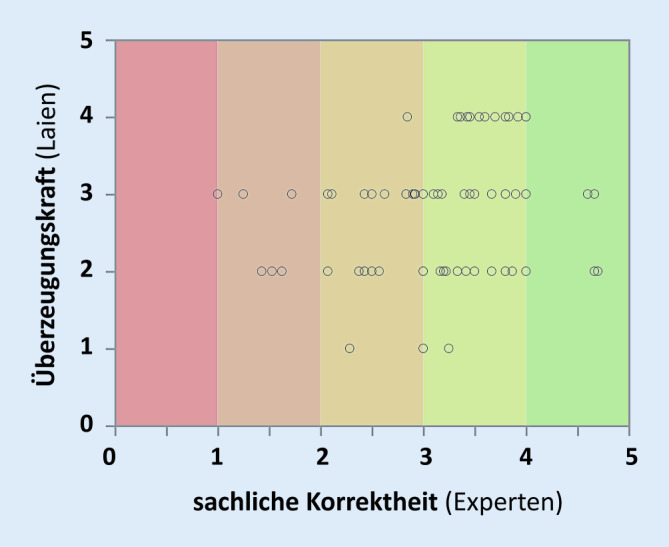

Nur eine schwache Assoziation bestand zwischen der Korrektheit der enthaltenen Aussagen in der Bewertung der Experten auf der einen Seite und der Überzeugungskraft des Gesamtinhalts in der Einschätzung der Laien auf der anderen Seite (Abb. 4). Die überzeugende Wirkung variierte deutlich, es zeigte sich nur eine mäßige Assoziation (Koeffizient 0,26, *p* < 0,017). Das Ausmaß an Überzeugungskraft wies keinen Zusammenhang mit objektiven Parametern wie Länge des Textes, Größe der Organisationseinheiten (Anzahl der tätigen Ärzte) oder Upload-Datum ins Netz auf, noch gab es einen Zusammenhang zwischen Aufrufen und den durch die Laien abgegebenen Bewertungen.

Vier Texte, die tatsächlich eine Art Komplikation oder Nebenwirkungen ansprachen, schnitten in der Tendenz besser ab als die große Mehrheit; angesichts der kleinen Gruppe und des explorativen Charakters wurden hier keine statistischen Kenngrößen benannt.

In den freien Kommentaren wurde wiederholt auf grammatikalische Fehler oder die schlechte Strukturierung der Informationen hingewiesen. Mehr als die Hälfte der befragten Laien gab im dafür vorgesehenen Freitextfeld offene Fragen an. Die meisten Rückfragen drehten sich um Risiken oder alternative Behandlungsmöglichkeiten, die in den Filmen nicht angesprochen wurden. Außerdem gab es zahlreiche Fragen und kritische Anmerkungen aufgrund der Verwendung offensichtlich unbekannter oder unklarer medizinischer Begriffe und Abkürzungen („optische Kohärenztomographie“, „Vitrektomie“, „Makuladegeneration“, „Aberrationen“, „IRPL“, „refraktive Chirurgie“), die in den Videos ohne Einführung oder Erläuterung verwendet worden waren.

### Formale Beurteilung

Die analysierten Texte hatte einen Umfang von im Median 256 Wörtern (Spanne: 107 bis 432). Gesprochen wurden somit im Median 2 Wörter pro Sekunde (Spanne: 1,1 bis 2,6 Wörter/s). Eine Mehrheit der Videos entsprach dem Charakter einer Vorstellung der Behandler (*n* = 19); 26 der 30 Videos stellten dabei gezielte Leistungen wie operative oder Lasereingriffe in den Mittelpunkt der Darstellung.

Es verwendeten 63 % der Videos (*n* = 19) mindestens einen inhaltlichen Superlativ (Tab. 2). Hier wurden Formulierungen wie „die oberflächlichsten Zellen“ nicht gewertet, aber alle Steigerungsformen mit attributivem Charakter zu einem Verfahren, Behandler oder Medizinprodukt. Am häufigsten wurden die Begriffe „neueste Technik“, „modernste Verfahren“ und „auf höchstem Niveau“ verwendet. Insgesamt wurden 33 Superlative gezählt.

**Table 2:** 

Verwendete Superlative	
*Modernste* Technik	*Neuesten* technischen Verfahren
Am *besten* geeignet	*Neueste* Geräte
Am *besten* therapieren	Auf *höchstem* Niveau
*Besten* Spezialisten	Zum *ersten* Mal in Ihrem Leben
Das *Beste* für Ihre Augen	*Bestens* ausgebildetes Mitarbeiterteam
*Modernste* Diagnostik	*Erste* Anlaufstelle
Medizin auf *höchstem* Niveau	*Neuesten* Stand
*Sicherste* Augenlasermethode	*Höchste* optische Güte
*Höchste* Sicherheit	*Modernste* Diagnostikgeräte
*Neuesten* medizinischen Standard	*Neueste* operative Verfahren
In *kürzester* Zeit	Weltweit *häufigste* Verfahren
*Neuartigste* Methode	*Sicherste* Verfahren
Augen haben *beste* Sicht verdient	*Neuesten* Therapiemethoden

Fünf der Videos benannten ein bestimmtes System oder Medizinprodukt, wobei einmal explizit der Herstellername „Wavelight“ (WaveLight GmbH, Erlangen, Deutschland) genannt wurde und in den anderen Fällen lediglich durch Nennung eines proprietär geschützten Namens eine unmittelbare Verbindung und eindeutige Zuordnung des Herstellers gegeben war: „E-Eye“ (E-swin, Linz, Österreich), „Evo Visian ICL“ (Staar Surgical, USA), „Smart Surf^ACE^“ (Schwind GmbH, Kleinostheim, Deutschland), „ReLEx® SMILE“ (Carl Zeiss Meditec, Oberkochen, Deutschland) und „2-RT™“ (Ellex Berlin, Deutschland). Allerdings beschränkten sich diese Verstöße gegen das Fremdwerbegebot auf nur 2 der insgesamt 20 verantwortlichen Urheber.

Ein Arzt warb mit einem „kostenlosen Eingriffs-Check“, was nach der gültigen Rechtsprechung die Ausnahmeregelung für kostenlose Auskünfte oder Ratschläge (§ 7 Abs. 1 HWG) ausdrücklich überdehnt [1]. Tatsächlich wurde von Laien positiv bewertet, was als Eignungsuntersuchung und Erstberatung vor einer Augenlaserbehandlung verboten ist (OLG Köln, 20.05.2016, Az.: 6 U 155/15; OLG München, 15.01.2015, Az.: 6 U 1186/14). Sämtliche Videos hielten sich an die Vorgaben, keine Preise für einzelne Leistungen zu nennen, was außerhalb der Praxisräume verboten ist, aber entsprechend der wirtschaftlichen Aufklärungspflicht innerhalb der Praxisräume erwünscht oder verpflichtend.

## Diskussion

Obwohl bereits eine Formulierung wie „Bei uns werden Sie bestens versorgt“ in einer sachlichen Information des Ärzteblatts über die Grenzen der Werbung als Beispiel für verbotene reißerische oder marktschreierische Mittel und nichtssagende oder reklamehafte Inhalte genannt wurde [1], waren entsprechende Superlative in fast zwei Drittel der untersuchten Videos aus der Augenheilkunde anzutreffen. Das spricht nicht nur für die Unkenntnis der berufsrechtlichen und juristischen Vorgaben. Offensichtlich steht auch die Motivation im Vordergrund, sich und das eigene Behandlungsangebot möglichst günstig darzustellen. Obwohl Augenchirurgen hier keine Ausnahme sind [14, 19–21], sind der Umfang und das Ausmaß an zumindest grenzwertigen Aussagen ernüchternd. Leider gab es in der untersuchten Stichprobe nur vereinzelte Ausnahmen, die Wissensvermittlung über Krankheiten, Beschwerden oder gesundes Verhalten im Interesse der Öffentlichkeit betrieben.

Der Wortlaut der Berufsordnung (MBO-Ä) lehnt sich an die Rechtsprechung des Bundesverfassungsgerichts an, nach der der „Schutz des Vertrauens der Patienten in die Integrität der Ärzteschaft im Interesse der Allgemeinheit“ liege (BVerfG, 01.06.2011, Az.: 1 BvR 233/10, 1 BvR 235/10). Die untersuchten Beispiele zeigen allerdings, dass Patienten nicht unbedingt darauf vertrauen können, dass – auch aus kommerziellen Interessen – eine korrekte und vollständige Information über Behandlungsmethoden und ihre Risiken stattfindet. Im Vordergrund der eingestellten Videos standen nahezu ausnahmslos Leistungen mit Eigenanteil oder besser honorierte operative Leistungen. Inhaltliche Informationen und Hintergründe zu relevanten Beschwerden oder häufigeren Erkrankungen wurden nicht thematisiert, sodass offensichtlich die Unterstützung oder qualitative Verbesserung der Selbstkontrolle und Patientenselektion im Hintergrund stand [22, 23]. Obwohl Ärzte in früheren Befragungen mehrheitlich eine ablehnende Haltung des Kundenbegriffs für Patienten und insbesondere auch gegenüber der Verengung der eigenen Position auf eine Verkäuferrolle berichteten [24], hatten einige der beschriebenen Videos wenig mit vollständiger und unabhängiger Beratung zu tun.

### Information statt Werbung fördert Gesundheitskompetenz

Im englischen Sprachgebrauch umfasst der Begriff „Health literacy“ die Motivation, das Wissen und die Kompetenz, Informationen für die Gesundheitsfürsorge, Krankheitsprävention und Gesundheitsförderung zu verstehen, zu bewerten und anzuwenden [25]. Obwohl der Erhalt des Sehsinns von Betroffenen meist über die Sorge um andere Beeinträchtigungen gestellt wird und chronische Erkrankungen der Netzhaut Selbstkontrolle und ein hohes Maß an Einsatz erfordern, wurden große Defizite an Wissen und Gesundheitskompetenz in Stichproben gefunden [26]. Die alarmierende Beobachtung kann viele Gründe haben wie eine unzureichende, wenig krankheitsorientierte Schulbildung oder die Vernachlässigung der ophthalmologischen Volkskrankheiten in der öffentlichen Wahrnehmung.

Die große Mehrheit der identifizierten Filme hat nicht die Verbesserung der Gesundheitskompetenz zum Ziel. Komplikationen wurden bis auf wenige Ausnahmen vollständig verschwiegen, der vermeintliche Vorteil von Leistungen übersteigert dargestellt. Dabei sollte nach allgemeinem Verständnis beachtet werden, Betroffenen ein sorgfältiges Abwägen zwischen möglichem Nutzen und den unweigerlichen Risiken zu ermöglichen [27]. Ohne Einblicke in relevante Nebenwirkungen, wie z. B. die Einbuße der Kontrastempfindlichkeit nach der Implantation einer diffraktiven Linse, bleibt eine Aufklärung unvollständig. Begriffe wie „Premiumlinse“ sollten nicht verwendet werden, erst recht wenn sie überhöht dem unsinnigen Kunstwort der „Kassenlinse“ gegenübergestellt wird. Moralisch ist es problematisch, eine einseitige Darstellung und positive Versprechungen zur Werbung möglicher Kandidaten zu nutzen, selbst wenn erst im zweiten Schritt vermeintliche Ausschlusskriterien und Risiken berücksichtigt würden. Wenn die Erwartungen – auch durch unzulässige Superlative – am Anfang falsch gesetzt werden, droht eine fehlgeleitete Übertherapie, z. B. indem wichtige Ausschlussgründe übersehen werden oder riskiert wird, relevante Vorbehalte aus der Patientenperspektive auszublenden.

### Rezeption und Zielgruppe

Angesichts der heterogenen Antworten muss die Einschätzung der Laien sehr vorsichtig interpretiert werden. Obwohl eine größere Stichprobe eine feinere Trennschärfe im Hinblick auf die Wirkung und Bewertung der Texte ermöglicht haben könnte, sind die kritische Aufnahme und umfangreiche Kommentierung ein Beleg für ein erhebliches Verbesserungspotenzial gegenüber der untersuchten Stichprobe. Auch ohne einen medizinischen Hintergrund kann man klare Erwartungen an die Qualität und die ethischen Grundanforderungen an Fachinformationen haben [28].

Gerade die umfangreiche Verwendung zahlreicher Fachtermini unterstreicht, dass die Urheber der Videos die Laienperspektive nicht berücksichtigt haben. Die Verständlichkeit hätte leicht verbessert werden können, z. B. indem ein Feedback im Konzeptstadium eingeholt wird. Fachwörter stehen zwar oft im Verdacht, die Attribution der vermeintlichen Kompetenz zu erhöhen; die nahezu einheitlich kritischen Rückmeldungen der jüngeren Gruppe von Laien lassen aber erwarten, dass dies angesichts einer zunehmend präferierten partizipativen Entscheidungsfindung kontraproduktiv ist [27].

Mundpropaganda zwischen Patienten, Klinikern und Ärzten und der Öffentlichkeit kann Einfluss nehmen, wo sich Patienten angesichts der freien Arztwahl in Deutschland vorstellen [29, 30]. Nicht allein die in der Stichprobe enthaltenen Privatpraxen meinten, Vorzüge ihrer Therapieangebote einseitig in den Vordergrund zu stellen. So legitim eine positive Darstellung des angebotenen Leistungsspektrums ist, so problematisch ist es, wenn die differenzierte Wirklichkeit und Notwendigkeit zur kritischen Indikationsstellung in den Hintergrund rückt und einseitig angepriesen wird. Mit dem Aufkommen des Internets und der mobilen Kommunikation ist es Patienten und Kollegen möglich, jederzeit zu recherchieren und Informationen mit einem Klick abzurufen [31]. Die Bewertung in Online-Portalen korreliert kaum oder gar nicht mit objektiven Parametern [32]; davon abgesehen gibt es klare Belege, dass die sichtbaren Noten stark mit dem finanziellen Engagement, also dem Verdienst des Portalbetreibers assoziiert sind. Gerade herausgenommene Negativbewertungen und selbst initiierte Bewertungen können das Ergebnis und Gesamtbild beeinflussen. Eine Recherche der Wochenzeitung *Die Zeit* zeigte ein erhebliches Verzerrungspotenzial des Arztbewertungsportals „Jameda“, einer Tochter des BURDA-Konzerns. Die Einordnung der Videos durch die Laien unterstreicht nochmals, wie eingeschränkt und heterogen das Urteil von Laien ist. Zumal auch Gütesiegel, Gesamtnote und z. B. die Focus-Liste, mit der auch eines der untersuchten Videos offensiv warb, den Anspruch suggerieren, die Behandlungsqualität abzubilden, müssen nicht nur die wirtschaftlichen Interessen auf dem zweiten Gesundheitsmarkt transparent gemacht werden. Betroffene können beurteilen, ob ein Arzt sich Zeit nimmt oder freundlich ist, dürften aber kaum die Qualität der Diagnosen oder Integrität der Ärzte kompetent bewerten können.

Die hier untersuchten Videos wurden ausnahmslos von den Ärzten selbst produziert und eingestellt. Dennoch bleiben – auch ohne vertragliche Kooperation oder finanzielle Unterstützung durch einen Hersteller von Medizinprodukten – die notwendige Grenze und kritische Distanz nicht gewahrt, wenn z. B. durch Daten nicht gestützte und somit falsche Versprechungen („*Damit können wir den Übergang in eine feuchte und damit die finale Form der AMD verhindern.*“) mit dem konkreten Gerät oder Hersteller verknüpft werden (§ 27 Abs. 3 S 4 MBO-Ä). Wenn Ärzte spezifische Geräte oder Hersteller nennen, stellt sich die Frage eines Verstoßes gegen das Fremdwerbeverbot (BVerfG, 01.06.2011, Az.: 1 BvR 233/10, 1 BvR 235/10). Gerade für sehr teure Geräte wie FEMTO-Laser und zugehörige Topografiegeräte dürften Ophthalmochirurgen nur in den seltensten Fällen mehrere konkurrierende Systeme vorhalten, deren Hersteller wiederum gewisse Alleinstellungsmerkmale für sich reklamieren („*Der Topolyzer™, der misst die Oberfläche mit einer Genauigkeit von 12.000 Punkten.*“).

Der Branchenwettbewerb für die Bereitstellung bestimmter Dienstleistungen kann dazu beigetragen haben, dass ein hoher Anteil der Problemvideos die refraktive Chirurgie bewarb. Eine amerikanische Studie fand eine deutliche Korrelation zwischen werbenden Aussagen und der Wahrscheinlichkeit späterer Haftpflichtfälle [33], und das sogar für Chirurgen mit sehr hohen Fallzahlen. Kein Augenarzt warb oder informierte über neuroophthalmologische oder entzündliche Erkrankungen. Trotz der ungleichen Verteilung der Subdisziplinen ist selbstverständlich eine faire Bewertung jedes einzelnen Kollegen unabdingbar. Obwohl Wettbewerb und das Bemühen um gute Medizin („Tue Gutes und spreche darüber“) als solches auch in Gesundheitsberufen nichts Schlechtes sind, müssen die juristischen Grenzen eingehalten werden. Das grundsätzliche Ziel von Werbung, Aufmerksamkeit und Interesse zu wecken, dürfen auch Ärzte verfolgen [31]. Die Grenze der Angemessenheit ärztlicher Information und Werbung ist allerdings klar überschritten, wenn die Darstellung Übertreibungen aufweist, aufdrängend oder gar belästigend wirkt. Wenn die Perspektive der Betroffenen in das Zentrum rückt, dürfte die Qualität profitieren. Deshalb sind Zuhören und die Beteiligung von Testpersonen angeraten, bevor Videos erstellt werden oder online gehen [34]. Außerdem sollten entsprechende Empfehlungen und Standards zur Patientenbeteiligung und Laienverständlichkeit stärker berücksichtigt werden [35–37]. Das bedeutet aber keinesfalls einen allzu großzügigen Umgang mit Testimonials [38]. Erfahrungsberichte bleiben immer Einzelfälle: Es ist zudem fraglich, ob es Interessierten hilft, wenn ein Augenarzt von der an ihm durchgeführten Laserbehandlung schwärmt. Außerdem sind sich Betroffene nicht immer alle der Auswirkungen einer Veröffentlichung im Internet bewusst.

### Limitationen

Verschiedene methodische Einschränkungen sind zu berücksichtigen. Obwohl die Stichprobe für die Suchbegriffe relativ umfangreich war, ist nicht auszuschließen, dass es für einzelne Krankheiten noch umfangreicheres und sachlich hochwertigeres Videomaterial über die identifizierten Filme hinaus gibt. Offensichtlich unterschieden sich die Videos in Bezug auf die Aufnahmequalität (Bild, Ton). Obwohl im Rahmen dieser Studie primär die Inhalte untersucht werden sollten, darf nicht vergessen werden, dass die Wirkung eines Videos oder die Beeinflussung aus einer Mischung aus optischen Eindrücken und Klangteppich beruht. Ton- und Bildkanal und daraus resultierende emotionale Wirkung wurden nicht ausgewertet. Dabei sind auch in der juristischen Beurteilung einer Werbemaßnahme nicht allein einzelne Worte oder Passagen des Werbetextes, sondern die Gesamterscheinung maßgeblich. Nicht berücksichtigt wurden Texte oder Domain-Namen der Homepages, z. B. ob entgegen den Vorgaben des Telemediengesetzes der Eindruck erweckt wurde, ein gewisses Fachgebiet abzudecken (z. B. www.ophthalmologie.de) oder als Einziger eine Fachrichtung vor Ort zu vertreten (www.augenberlin.de).

Ausreißer durch Einzelmeinungen sollten durch die Unterteilung und Variation der Reihenfolge vermindert werden. Dennoch ist es nicht auszuschließen, dass die Videos teilweise im Vergleich zueinander bewertet wurden, was angesichts einiger Ausprägungen dazu geführt haben könnte, dass der Gesamtschnitt nach oben oder unten gedrückt worden sein könnte. Die Beobachtung konnte außerdem nur eine Momentaufnahme zu einem Zeitpunkt darstellen.

Der Anteil der faktischen Aussagen schwankte zwischen den Videos trotz eines vergleichbaren Textumfangs erheblich. So wurde in der Planung der Untersuchung vermutlich unterschätzt, dass ein Großteil der Aussagen persönliche oder kaum zu überprüfende Behauptungen enthalten, wie das Prahlen mit erreichten Operationszahlen, die vorgehaltene Expertise, verwendete Gerätetechnologien oder das Angehören einer Kommission. Diese Aussagen konnten selbst von den Experten letztlich nicht auf ihren Wahrheitsgehalt überprüft werden.

Die Diskrepanz in der Expertenbeurteilung unterschied sich für die Einordnung der Videos insgesamt weniger als die Einordnung der Einzelaussagen. Möglicherweise war einerseits die Trennschärfe der Kategorien (Plausibilität, Klarheit, Relevanz, Vollständigkeit) ungleichmäßig definiert, d. h. die Experten unterschieden sich in ihrem subjektiven Erwartungshorizont. Um Beeinflussung zu vermeiden, wurde auf eine vorherige Absprache oder zusätzliche Harmonisierung im Vorfeld verzichtet. Auch die begrenzte Zahl der Experten war für die Streuung verantwortlich. Vermutlich wäre mit einer größeren Anzahl von Bewertungen eine noch deutliche Abstufung unterschiedlicher Qualitäten möglich gewesen. Aus testtheoretischer Sicht ist aber zu beachten, dass eine hohe Übereinstimmung für einen wesentlichen Anteil der untersuchten Videos mit mehrheitlich offensichtlich falschen Aussagen weniger zur Trennschärfe beitragen kann als das Aufsplittern der Skala für Videos mit uneinheitlich bewerteten Aussagen. Die größte Übereinstimmung bestand eindeutig in Bezug auf die Unvollständigkeit der Informationen. In Bezug auf die Plausibilität und Klarheit hat eine einzelne von allen Experten als falsch identifizierte Aussage nicht dazu geführt, dass die Plausibilität des gesamten Videos die niedrigste Bewertung erhielt.

### Herausforderungen für die Zukunft

Obwohl einige der beschriebenen Beispiele – nicht nur nach Einschätzung der jeweiligen Experten – den Tatbestand einer strafbaren Handlung erfüllen dürften, ist den Autoren bestimmt nicht an einer pauschalen Verunglimpfung der jeweiligen Kolleginnen und Kollegen gelegen. Gerade die Rückmeldung und Bewertung der befragten Laien zeigen, dass aber der Schutz von Patienten aktuell noch unzureichend ist. Viele der Formulierungen suggerieren offensichtlich falsche Annahmen, wie z. B. die Aussage, ein unterschwelliger Nanosekundenlaser könne die neovaskuläre Makuladegeneration „*verhindern*“ [39], jede Katarakt müsse *„so früh wie möglich“* operiert werden [40], die Vitreolyse garantiere das „*vollständige Verschwinden*“ von Trübungen [41] oder diffraktive bzw. gelbe Linsen versprächen „*eine bessere Abbildungsqualität*“ [42, 43]. Weil aktuell mit der höchsten Evidenzstufe weder genereller Vorteil noch Nutzen der Kataraktoperation mittels Femtosekundenlaser gefunden wurde [44], ist es falsch, ein steinzeitlich verzerrtes Gegenbild der konventionellen Chirurgie beispielsweise mit der Verwendung eines groben und ungenauen „Skalpells“ zu konstruieren.

Es gab keinen Zusammenhang zwischen der Einschätzung der Laien und der Bewertung von Experten. Offensichtlich ist aber keine zuständige Aufsichtsbehörde aktiv, die falschen Unterstellungen widerspricht oder proaktiv und ohne konkretes Verdachtsmoment hochgeladene Videos beobachtet. Patientensicherheit darf aber nicht erst dann ins Blickfeld rücken, nachdem es bereits zu Körperverletzungen gekommen sein sollte. Das Ausblenden von Risiken und die Verwendung von obsoleten Begriffen wie „Brillenfreiheit“ müssen aufmerken lassen.

Nicht zuletzt darf man die Außenwirkung nicht unterschätzen [45]. Am Ende des Tages signalisieren die erhobenen Rückmeldungen, dass die Videos der Augenpraxen als Eigenwerbung durchschaut werden. Das dürfte also auch nicht unbedingt vorteilhaft zum Bild der Fachgruppe beitragen [46]. Curtis Margo warnte bereits 1988 vor den nachteiligen Auswirkungen kommerziell ausgerichteter Werbung auf die ärztliche Autonomie und einem entsprechenden Vertrauensverlust [47]. Eigentlich erschließen sich keine Gründe, wieso Text, Bild und Videomaterialien nicht bereits vor der Veröffentlichung einer fachlichen Prüfung durch unabhängige Vertreter einer Fachgesellschaft unterzogen werden, ohne dass hohe Gebühren oder finanzielle Anreizsysteme andere Prioritäten bedeuten. Wenn entsprechende Qualitätsmaßstäbe Verbreitung finden, muss nicht unbedingt ein Maß an Überregulierung erfolgen [12].

Keinesfalls sollte die Chancen des Internets unter Generalverdacht gestellt werden [48]. Eigentlich besteht ein hohes Maß an Eigeninteresse, dass in Zukunft differenziertere Informationen angeboten werden. Unabhängige Einrichtungen wie das IQWIG oder die Selbsthilfe bieten hier zwar ein Angebot von Texten [49]. Dennoch dürfte das Potenzial, das Verständnis von Betroffenen mittels attraktiven Bildmaterials zu verbessern, bei Weitem noch nicht ausgeschöpft sein. Attraktive und verständliche Filme helfen nicht nur der Wissensvermittlung gegenüber Analphabeten [50–52]. Unabhängige Organisationen wie die Initiativgruppe „Früherkennung diabetischer Augenerkrankungen“ (IFDA) bieten attraktives Bildmaterial, Videos und Animationen an [53].

Wenn Augenärztinnen und Augenärzte sich engagieren und für das Erstellen und Hochladen von bewegten Bildern Geld und Zeit investieren, sind sie in Zukunft auch stärker gefordert, die Perspektive von Betroffenen nicht zu vergessen. Früher waren fremdsprachliche Bezeichnungen oder unverständliche Begriffe nach dem Heilmittelwerbegesetz verboten, eben um den inflationären Gebrauch zur Profilierung und Herausstellung vermeintlicher Fachkompetenz auf Kosten der Verständlichkeit zu verhindern. Nüchtern muss festgestellt werden, dass die jetzt wieder mögliche Verwendung von Fachbegriffen der Klarheit nicht unbedingt gutgetan hat. Wenn Ärzte es nicht schon aus ihrem Alltag her besser wissen, die Inhalte ihres Fachgebiets verständlich und einfach zu erklären, sollten sie dennoch aktiver ein Feedback einholen, welche Botschaft den Adressaten erreicht hat („Teach back“) [54]. Eine gute Patientenversorgung sollte immer darauf beruhen, dass Beratung und Behandlung im Interesse der Patienten erfolgen. Gerade die notwendige Beschränkung auf eine begrenzte Zeit der Aufmerksamkeit potenzieller Konsumenten macht eine differenzierte Gegenüberstellung von Möglichkeiten und Risiken der Behandlungsoptionen zu keiner einfachen Herausforderung.
